# Health-Related Quality of Life Profiles in Dialyzed Patients With Varying Health Literacy. A Cross-Sectional Study on Slovak Haemodialyzed Population

**DOI:** 10.3389/ijph.2021.585801

**Published:** 2021-04-14

**Authors:** Ivana Skoumalova, Andrea Madarasova Geckova, Jaroslav Rosenberger, Maria Majernikova, Peter Kolarcik, Daniel Klein, Andrea F. de Winter, Jitse P. van Dijk, Sijmen A. Reijneveld

**Affiliations:** ^1^ Department of Community and Occupational Medicine, University Medical Center Groningen, Groningen, Netherlands; ^2^ Department of Health Psychology and Research Methodology, Faculty of Medicine, Pavol Jozef Safarik University, Kosice, Slovakia; ^3^ Graduate School Kosice Institute for Society and Health, Faculty of Medicine, Pavol Jozef Safarik University, Kosice, Slovakia; ^4^ Olomouc University Social Health Institute, Palacky University, Olomouc, Czech Republic; ^5^ FMC-Dialysis Services Slovakia, Kosice, Slovakia; ^6^ Institute of Mathematics, Faculty of Science, Pavol Jozef Safarik University, Kosice, Slovakia

**Keywords:** health-related quality of life, dialyzed patients, chronic kidney disease, health literacy, CKD-5

## Abstract

**Objectives:** Chronic kidney disease (CKD) strongly affects patients’ health-related quality of life (HRQoL), mostly in the advanced stages of CKD. Health literacy (HL) may affect this association, in particular for some aspects of HRQoL. The aim of this study is to compare the profiles of HRQoL in dialyzed patients with varying HL.

**Methods:** We obtained data on HL using the Health Literacy Questionnaire (HLQ) and on HRQoL using the Kidney Disease Quality of Life – Short Form (KDQoL-SF 1.3) in a multicentre cross-sectional study in 20 dialysis clinics in Slovakia (*n* = 542; mean age = 63.6 years; males: 60.7%). We compared HRQoL for three HL groups using ANOVA and the Kruskal-Wallis test.

**Results:** Patients with low HL reported worse HRQoL than patients with moderate and high HL. The greatest differences between HL groups were found in the scales Effect of kidney disease, Cognitive function, Quality of social interaction, Social support, Dialysis staff encouragement, Patient satisfaction, Physical functioning, Pain, Emotional well-being and Social function. *p*-values in all cases were <0.001.

**Conclusion:** Patients with low HL have a worse HRQoL in several domains than patients with a higher HL. Increasing HL capacities and better supporting patients with low HL should thus be given priority to support their HRQoL and at least maintain its level.

## Introduction

Chronic kidney disease (CKD) is a major public health problem that causes a large share of cardiovascular and all-cause mortality and morbidity worldwide [1–3]. CKD represents a great burden for the health care system and thus for public expenditures, as well [4, 5]. CKD is without symptoms until it has progressed to later stages, especially to end-stage renal disease (ESRD) when dialysis or renal replacement is needed to maintain a patient’s life [6]. In this stage, the disease affects patient’s life considerably including the patient’s physical functioning, mental health and his/her social life. Moreover, dialyzed patients are under constant medical supervision; they attend dialysis frequently and have to adhere to various recommendations by health professionals regarding diet, fluid intake and medications, which creates an extra burden on their daily life [7]. Therefore, maintaining a good quality of life is an important part of treatment for CKD patients.

Advanced CKD strongly affects a patient’s health-related quality of life (HRQoL) due to both the disease and its treatment [8–11]. Poor HRQoL is common in ESRD patients and is associated with increased morbidity and mortality [12, 13]. HRQoL comprises a wide range of aspects in accordance with the biopsychosocial model of health and illness [14, 15]: physical aspects (symptoms of the disease and its effects on everyday life, work and responsibilities), mental aspects (perceived burden of the disease and emotional well-being) and social aspects related to illness (quality of social interaction, social functioning and social support from relevant others and health care providers).

Several factors may affect the deterioration of HRQoL in CKD patients, and one of these is health literacy (HL) [16]. HL is defined as a person’s knowledge, motivation and competence to access, understand, appraise and apply health information in order to make judgments and decisions in their everyday life concerning health care, disease prevention and health promotion to maintain or improve their quality of life [17]. Patients with limited HL may fail to understand and apply health information for appropriate self-care activities, and limited HL may highly affect dialyzed patients, who have to adhere to strict recommendations regarding diet, medications and dialysis and must effectively cooperate with healthcare providers. These challenges may in turn negatively affect the quality of their physical health, mental health and social life [18–21].

HL may affect HRQoL in dialyzed patients, but evidence on this is scarce. Previous research [18, 22] showed that dialyzed patients with limited HL reported worse HRQoL than patients with better HL. In contrast Green et al. [23], using a one-dimensional tool for assessing HL (REALM), did not find an association of HL and QoL in dialyzed patients. However, evidence is fully lacking regarding more detailed aspects of HRQoL in dialyzed patients with varying HL, and this may be important for tailoring care in order to maintain HRQoL in dialyzed patients. Therefore, the aim of our study is to compare the HRQoL profiles of dialyzed patients with varying HL.

## Methods

### Sample and Procedure

We collected data from January 2018 to November 2018 within a network of 20 dialysis clinics belonging to one private network (FMC-dialysis services Slovakia) in Slovakia (covering about 20% of the total Slovak dialysis population). This dialysis therapy is fully reimbursed by a compulsory health insurance system. Inclusion criteria were age over 18 years, a diagnosis of CKD-5 and undergoing haemodialysis treatment for at least 90 days. Exclusion criteria were an inability to fill in the questionnaire (due to dementia or mental retardation, inability to read the Slovak language) and acute severe intercurrent illness, obtained from medical records. All patients who met the eligibility criteria were asked to participate in the study.

Data were obtained by questionnaires filled in by patients during their routine visits to the dialysis clinic. Patients agreed to participate in the study by signing an informed consent prior to the study. They then filled in the questionnaires using tablets, with data recorded to an online platform with full confidentiality assured by means of a personal identification code. A research assistant was available for technical support.

We included 567 patients on maintenance haemodialysis (70.1% of those approached), 25 of whom were excluded due to not filling in the questionnaire related to HL (*n* = 9) or missing too many items in it (*n* = 16), leading to a final sample of 542 patients.

### Ethics

The study was approved by the Ethics Committee of the Faculty of Medicine of P.J. Safarik University (15N/2017) and the Ethics Committee of FMC-dialysis services (November 23, 2017). All data were collected in accordance with the ethical standards as laid down in the 1964 Declaration of Helsinki and its later amendments or comparable ethical standards.

### Measures

*Health-related quality of life* was measured by the Kidney Disease Quality of Life – Short Form, version 1.3 (KDQoL-SF™, [10]). This is a self-report measure developed for CKD patients and those on dialysis and is widely used in research [24, 25]. It consists of 43 kidney disease targeted items within eight scales and three additional quality of life scales. These scales are: Symptom Problem Scale (SPS), Effects of Kidney Disease (EKD), Burden of Kidney Disease (BKD), Work Status Scale (WSS), Cognitive Function Scale (CFS), Quality of Social Interaction Scale (QSIS), Sexual Function Scale (SXFS), Sleep Scale (SS), Social Support Scale (SSS), Dialysis Staff Encouragement Scale (DSES) and Patient Satisfaction Item (PSI). The second part of the questionnaire is the 36-item health survey (SF-36) consisting of eight scales: Physical Functioning Scale (PFS), Role – Physical Scale (RPS), Pain Scale (PS), General Health Scale (GHS), Emotional Well-being Scale (EWS), Role – Emotional Scale (RES), Social Function Scale (SFS) and Energy Fatigue Scale (EFS). The scales score ranges from 0 to 100. A higher score reflects a better quality of life. The Cronbach’s Alpha in our sample ranges from 0.43 to 0.94. We did not include the Symptom Problem Scale for peritoneal dialysis, as our sample consists only of haemodialyzed patients. See **Appendix A** for more information on the measurement tool.

*Health Literacy* (HL) was measured using the Slovak version of the Health Literacy Questionnaire (HLQ, [26]), a multidimensional tool [27] consisting of nine domains of HL related to accessing, understanding and using information to make decisions about health (Cronbach’s Alpha in our sample ranges from 0.77 to 0.90). A higher mean score in a particular domain indicates better HL in that domain [27]. We categorized this measure using hierarchical cluster analysis [28] on standardized z-scores of all HL domain, leading to clusters of cases with similar HL characteristics. This method minimizes the within-cluster variance in a stepwise manner leading to clusters that are as different as possible. Three clusters were used for further analyses (low HL group, moderate HL group, high HL group), representing different levels of HL consistently across all domains in a particular cluster. The mean HLQ score for the nine domains of the three HL groups of patients are described in Table 1.

**TABLE 1:** HLQ mean scores of patients in three health literacy groups (hierarchical cluster analysis, patients from 20 dialysis clinics, Slovakia 2018, *n* = 542).

	Low HL group			Moderate HL group			High HL group		
	95% CI			95% CI			95% CI		
HLQ domain	M	Lower	Upper	M	Lower	Upper	M	Lower	Upper
Feeling understood and supported by health care providers^a^	2.94	2.87	3.00	3.21	3.17	3.25	3.89	3.85	3.94
Having sufficient information to manage my health^a^	2.76	2.70	2.82	3.15	3.12	3.18	3.83	3.77	3.89
Actively managing my health^a^	2.77	2.71	2.83	3.08	3.05	3.12	3.66	3.58	3.74
Social support for health^a^	2.91	2.85	2.98	3.25	3.21	3.29	3.83	3.78	3.89
Appraisal of health information^a^	2.55	2.47	2.63	3.00	2.96	3.04	3.33	3.19	3.47
Ability to actively engage with health care providers^b^	3.32	3.23	3.42	4.01	3.97	4.06	4.75	4.69	4.82
Navigating the health care system^b^	3.04	2.95	3.14	3.86	3.81	3.91	4.58	4.48	4.67
Ability to find good health information^b^	3.15	3.06	3.24	3.91	3.86	3.97	4.63	4.56	4.70
Understand health information well enough to know what to do^b^	3.26	3.16	3.35	3.95	3.90	4.00	4.52	4.45	4.60

a Mean score ranges from 1 to 4.

b Mean score ranges from 1 to 5.

Sociodemographic data were measured using the questionnaire and included gender, education (lower education: elementary education and apprenticeship vs. higher education: high school and university), marital status (with partner vs. without a partner) and living conditions (living alone vs. with family member/s). We compared patients in productive age (≤50) with those in late productive and post-productive age (>50) as this might be associated with their social participation.

### Statistical Analyses

First, we assessed the sociodemographic characteristics of the sample and the three HL groups. Second, we assessed associations between the HL groups and HRQoL (continuous level) using one-way ANOVA and the Kruskal-Wallis test. The statistical significance of differences between the HL groups was tested using the post hoc Bonferroni tests in the case of ANOVA and Dunn’s tests with Bonferroni correction in the case of Kruskal-Wallis tests. Statistical analyses were performed using SPSS v. 23.0 for Windows [29].

## Results

### Baseline Characteristics

Of the 542 patients (mean age 63.6 years, standard deviation = 14.12), most were men (61%) and most older than 50 years (82%); almost half of the patients had a lower education (49%) and were without a partner (42%), and 18% of patients lived alone (Table 2).

**TABLE 2:** Sociodemographic characteristics of the sample and three health literacy groups (patients from 20 dialysis clinics, Slovakia 2018, *n* = 542).

	Total sample	Low HL group	Moderate HL group	High HL group		
Characteristics	*n* (%)	*n* (%)	*n* (%)	*n* (%)	Difference between HL groups	*p*-value^a^
Health literacy (total)		172 (31.7)	293 (54.1)	77 (14.2)		
Gender						ns
Male gender	329 (60.7)	105 (61.0)	181 (61.8)	43 (55.8)		
Age						ns
>50 years	444 (81.9)	147 (85.5)	237 (80.9)	60 (77.9)		
Education						ns
Lower education	266 (49.1)	92 (53.5)	139 (47.4)	35 (45.5)		
Marital status^b^					Low HL group vs. Moderate HL group	0.004
Without partner	223 (41.5)	83 (49.1)	105 (36.1)	35 (45.5)		
Living conditions^c^					Low HL group vs. Moderate HL group	0.004
Living alone	94 (17.8)	42 (25.0)	41 (14.3)	11 (14.9)		

a *p*-values are for comparison of categories of each variable by health literacy using Pearson’s chi-square test.

b Missing data = 5.

c Missing data = 14.

### HRQoL Profiles of Dialyzed Patients With Different HL

**FIGURE 1:**
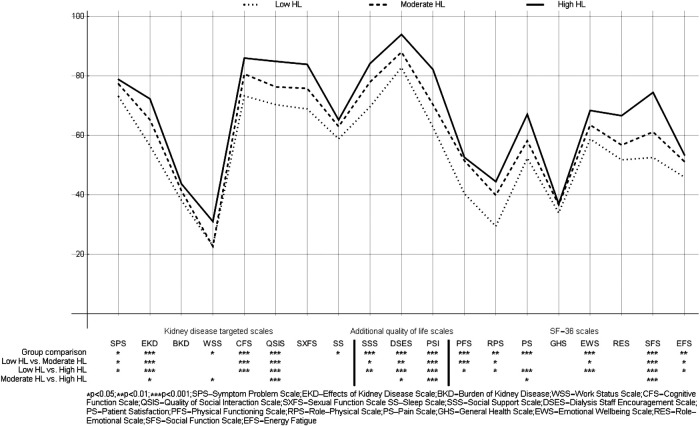
Health-related quality of life profiles in three groups of dialyzed patients with varying levels of health literacy (patients from 20 dialysis clinics, Slovakia 2018, *n* = 542).

We found HRQoL to differ between the three HL groups in 15 out of the 19 HRQoL scales (Table 3). Post hoc comparisons (Figure 1) revealed that low HL patients had a worse HRQoL than moderate and high HL patients. Moreover, patients in the moderate HL group had a worse HRQoL than the high HL group for seven HRQoL scales. The greatest differences between the three HL groups regarded the scales EKD, CFS, QSIS, SSS, DSES, PSI, PFS, PS, EWS and SFS.

**TABLE 3:** Differences in three health literacy groups in health-related quality of life scales (ANOVA and Kruskal-Wallis test, patients from 20 dialysis clinics, Slovakia 2018, *n* = 542).

	Low HL group Mean (SD)	Moderate HL group Mean (SD)	High HL group Mean (SD)	Anova/Kruskal-Wallis*
Kidney disease targeted scales^a^				
Symptom problem	73.12 (16.48)	77.34 (14.37)	78.85 (14.43)	F (2, 442) = 4.48, *p* = 0.012
Effects of kidney disease	56.57 (22.03)	65.27 (20.63)	72.32 (18.38)	F (2, 473) = 15.92, *p* < 0.001
Burden of kidney disease	38.18 (23.53)	41.43 (24.36)	43.75 (27.13)	F (2, 528) = 1.60, *p* = 0.203
Work status*	22.84 (31.08)	22.68 (34.05)	31.08 (32.80)	H (2) = 6.009, *p* = 0.050
Cognitive function	73.33 (19.87)	80.70 (18.25)	86.05 (14.73)	F (2, 523) = 14.77, *p* < 0.001
Quality of social interaction	70.38 (17.60)	76.36 (17.96)	84.94 (13.95)	F (2, 530) = 19.02, *p* < 0.001
Sexual function*	68.94 (29.86)	75.84 (26.17)	83.93 (17.29)	H (2) = 2.675, *p* = 0.262
Sleep	58.87 (20.00)	63.02 (18.71)	65.24 (20.92)	F (2, 520) = 3.53, *p* = 0.030
Additional quality of life scales^b^				
Social support	69.70 (30.17)	78.01 (29.82)	84.23 (27.01)	F (2, 512) = 7.10, *p* = 0.001
Dialysis staff encouragement	82.88 (19.68)	88.07 (15.40)	94.00 (17.60)	F (2,523) = 11,48, *p* < 0.001
Patient satisfaction	62.83 (19.68)	70.40 (20.00)	82.22 (19.63)	F (2, 527) = 24.13, *p* < 0.001
SF-36 scales^c^				
Physical functioning	40.46 (31.56)	51.52 (29.45)	52.64 (31.25)	F (2, 511) = 7.72, *p* < 0.001
Role – physical^d^	29.46 (39.86)	39.91 (41.07)	44.48 (45.09)	F (2,532) = 4.77, *p* = 0.009
Pain	52.40 (26.78)	58.24 (26.38)	67.13 (27.22)	F (2, 528) = 8.10, *p* < 0.001
General health	33.96 (15.04)	36.67 (15.85)	36.95 (16.84)	F (2, 517) = 1.74, *p* = 0.177
Emotional well-being	58.83 (17.00)	63.55 (18.87)	68.43 (19.64)	F (2, 510) = 7.47, *p* = 0.001
Role – emotional^e^	51.81 (45.02)	56.79 (44.50)	66.67 (43.89)	F (2, 526) = 2.90, *p* = 0.056
Social function	52.59 (21.52)	61.21 (25.62)	74.49 (23.45)	F (2, 516) = 21.45, *p* < 0.001
Energy fatigue	45.97 (19.30)	51.22 (20.50)	53.45 (19.95)	F (2, 519) = 4.94, *p* = 0.007

a Missing data for particular scale ranges from 9 to 97, except of Sexual function scale (missing = 399).

b Missing data for particular scale ranges from 12 to 27.

c Missing data for particular scale ranges from 7 to 29.

d Role limitations due to physical problems.

e Role limitations due to emotional problems.

## Discussion

We found that HRQoL is poorer in dialyzed patients with lower HL. Our detailed analyses of physical, mental and social aspects of HRQoL has allowed us to identify the most problematic areas of HRQoL. To our knowledge, this is the first study to use a detailed HRQoL profile of dialyzed patients with varying HL. We found that patients with lower HL suffer from worse HRQoL than patients with moderate or higher HL. This confirms the findings of Dodson et al. and Stømer et al. [18, 22], although they used a different measurement tools for assessing HRQoL and assessed less domains of HRQoL. The current study provided a better understanding of the impact of HL on different domains. The greatest differences between the three HL groups regarded the scales related to the impact of kidney disease on the patient's functional health and emotional status (EKD, PFS, PS, EWS). Patients with lower HL also scored lower in the scales related to the social dimension of their quality of life (QSIS, SSS, SFS) and in the scales related directly to their relation with dialysis staff and their satisfaction with medical care (DSES, PSI). An explanation of these findings may be that patients with lower HL have problems with understanding health information or are unable to handle complex tasks regarding their diet and medications or to communicate effectively with health care providers [30]. Subsequent failure in meeting the demands related to self-care, self-management [31], engagement in treatment or in cooperation with health care providers may result in a poorer HRQoL regarding their functional status, quality of social life with relevant others and also the engagement with health care providers, which may be crucial for effective treatment.

We did not find significant differences between the three HL groups regarding some aspects of HRQoL, such as the Burden of kidney disease (BKD), Sexual function scale (SXFS), General health scale (GHS) and Role – emotional scale (RES). An explanation may be that some of these aspects are not related to HL capacities, such as sexuality or role limitations due to emotional problems. As regards the perceived burden of the disease and general health scale, we found that in these two aspects patients in our sample scored the lowest, regardless of their level of HL. This may indicate that health literacy capacities cannot compensate for the overall negative effect of the disease on their health and their perceived burden due to the disease. We found that patients with low HL were more likely to live alone and were more likely to live without a partner than patients with moderate HL. These findings are partly consistent with the findings of Geboers et al. [32] who found associations between low HL and loneliness, being engaged in social activities and having social contacts, but did not found associations between low HL and living conditions (living alone vs. living with others). Thus patients with limited HL may be even more vulnerable and requiring more support to be able to manage their health condition. We didn’t find associations of age, gender and education level with HL. This is partly inconsistent with the findings of other studies focusing on haemodialyzed patients, in which a lower level of education [23] and also male gender [20, 33] were significantly associated with limited HL.

### Strengths and Limitations

The major strengths of our study regard the representativeness of our sample, which covered ESRD patients undergoing haemodialysis in 20 dialysis clinics in Slovakia, and the relatively high response rate (70%). The use of a disease-specific HRQoL-related questionnaire (KDQoL – SF) as well as the generic core for QoL (SF-36) enabled us to bring detailed information on HRQoL profiles in a homogenous group of dialyzed patients.

Our study has some limitations as well. As the study had a cross-sectional design, we are unable to make causal inferences. Furthermore, the data are self-reported, which can result in some social desirability and thus in some information bias. Using self-report questionnaires may have led to some selection bias, with people with very low (health) literacy excluded, and thus to some underestimation of the real differences. Finally, some scales (WSS, QSIS and GHS) of this Slovak version showed a lower internal consistency than was found in the validation studies of the English original [10]. This may have added some measurement error, and thus an underestimation of the associations. This also requires further study on e.g., the impact of cultural factors and the results of our research should be interpreted with caution regarding the scales mentioned, as we used the best method to translate, i.e., forward-backward*.*

### Implications

Our findings that patients with low HL show worse HRQoL than patients with higher HL suggest that it is important to support patients with limited HL to maintain their HRQoL. Such support could relate to their capacities to understand, appraise and adequately use relevant health information to secure proper adherence to treatment and good cooperation and communication with health care providers. Another way of helping may be to offer psychological support for better coping with the disease. In addition, the responsiveness of the health care system to the needs of low HL patients may be improved [34, 35]. This may include effective patient-centred care taking into account specific barriers and limitations connected with low HL [31].

In future research it will be important to study the mechanisms responsible for the association between health literacy and HRQoL. Insight into the role of potential mediators, such as lifestyle, medication adherence, perceived control or the quality of the communication by health care professionals, will support the improvement of interventions aimed at maintaining HRQoL in dialyzed patients.

### Conclusion

We found that dialyzed patients differed in the HRQoL profile according to the level of their HL. Recognizing HL needs and limitations in dialyzed patients and tailoring care and health related communication towards those with low HL may help improve their HRQoL.

## Data Availability

The raw data supporting the conclusions of this article will be made available by the authors, without undue reservation.

## APPENDIX A

**Table:** Detailed information on Kidney Disease Quality of Life – Short Form (KDQoL‐SF^TM^), Version 1.3

	**Number of items**	**Cronbach's Alpha**
**Kidney disease targeted areas**		
Symptom problem scale haemodialysis	12	0.841
Symptom problem scale peritoneal	n/a	
Effects of kidney disease scale	8	0.844
Burden of kidney disease scale	4	0.741
Work status scale	2	0.427
Cognitive function scale	3	0.804
Quality of social interaction scale	3	0.460
Sexual function scale	2	0.897
Sleep scale	4	0.622
**Additional quality of life scales**		
Social support scale	2	0.812
Dialysis staff encouragement scale	2	0.844
Patient satisfaction item	1	n/a
**SF‐36**		
Physical functioning scale	10	0.937
Role physical scale^a^	4	0.884
Pain scale	2	0.882
General health scale	5	0.544
Emotional well-being scale	5	0.723
Role emotional scale^b^	3	0.888
Social function scale	2	0.689
Energy fatigue scale	5	0.781

^a^ Role limitations due to physical problems.

^b^ Role limitations due to emotional problems.
